# The First Evaluation of Proteinase K-Resistant Prion Protein (PrP^Sc^) in Korean Appendix Specimens

**DOI:** 10.3390/medicina58070947

**Published:** 2022-07-18

**Authors:** Sae-Young Won, Yong-Chan Kim, Yu-Ni Lee, Chan-Gyun Park, Woo-Young Kim, Byung-Hoon Jeong

**Affiliations:** 1 Korea Zoonosis Research Institute, Jeonbuk National University, Iksan 54531, Jeonbuk, Korea; gkfh32@jbnu.ac.kr (S.-Y.W.); kych@jbnu.ac.kr (Y.-C.K.); 2 Department of Bioactive Material Sciences, Institute for Molecular Biology and Genetics, Jeonbuk National University, Jeonju 54896, Jeonbuk, Korea; 3 Presbyterian Medical Center, Jeonju 54987, Jeonbuk, Korea; nnenn02@naver.com (Y.-N.L.); pckyun@hanmail.net (C.-G.P.)

**Keywords:** prion, PrP^Sc^, Creutzfeldt–Jakob disease, variant CJD, PRNP, appendectomy, mutation, polymorphism

## Abstract

*Background and Objectives:* Prion diseases are fatal neurodegenerative disorders caused by the abnormal proteinase K-resistant prion protein (PrP^Sc^). Since variant Creutzfeldt–Jakob disease (CJD) was first reported in the United Kingdom (UK) in 1996, the occurrence of variant CJD has been reported in over 10 countries. To date, variant CJD has not been reported in Korea. However, the E211K somatic mutation in the prion protein gene (*PRNP*), which is related to bovine spongiform encephalopathy (BSE), was reported in Korean Holstein cattle, and atypical BSE, which is supposed to be sporadic BSE, has been occurring in many countries, including Japan and the USA. These results suggest that BSE may occur naturally in Korea. Thus, we performed a preemptive PrP^Sc^ test in appendix specimens to diagnose variant CJD in a Korean population*. Materials and Methods:* In the present study, we investigated CJD-related mutations and polymorphisms of the *PRNP* gene and carried out an examination on PrP^Sc^ in appendix specimens of Korean patients after appendectomy. *Results:* In all Korean appendix specimens tested, PrP^Sc^ bands were not detected. *Conclusion:* To the best of our knowledge, this was the first evaluation of PrP^Sc^ in Korean appendix specimens.

## 1. Introduction

Prion diseases, also known as transmissible spongiform encephalopathies (TSEs), are fatal neurodegenerative disorders affecting the brain in humans and in animals [1]. In humans, prion diseases are classified as Creutzfeldt–Jakob disease (CJD), fatal familial insomnia (FFI), and Gerstmann–Sträussler–Scheinker syndrome (GSS) [2]. The major form of human prion disease is CJD, which is subdivided into sporadic, familial, variant, and iatrogenic CJD [3]. Although the exact cause of sporadic CJD is not known, somatic mutation in the prion protein gene (*PRNP*) has been considered one of its causal factors [4,5]. In addition, genetic polymorphisms of human *PRNP*, including M129V and E219K, play a pivotal role in susceptibility to sporadic and variant CJD [6]. M129V and E219K heterozygotes showed a protective effect to sporadic CJD. Familial CJD is caused by germline mutations in the human *PRNP* gene, including G114V, D178N, V180I, T183A, T188K, E196K, E200K, V203I, R208H, V210I, E211Q, M232R, and P238S [7].

Iatrogenic CJD is transmitted via contaminated surgical instruments, corneal transplants, and blood transfusions derived from patients with prion disease [8]. Among the several subtypes of CJD, variant CJD is the only zoonotic prion disease that is transmitted from animals, and it is known to be caused by the consumption of meat contaminated with bovine spongiform encephalopathy (BSE) [9]. Since variant CJD is caused by PrP^Sc^ contaminated meat intake, PrP^Sc^ accumulation has been observed in the appendix and in the tonsil tissues of variant CJD patients [10]. Thus, PrP^Sc^ detection in these tissues is useful for the diagnosis of variant CJD.

In Korea, 30 to 40 cases of CJD have been reported annually, and 12.8% of CJD cases were under 49 years of age between 2016 and 2019. However, variant CJD has not been officially reported in Korea. In a previous study, in the UK, abnormal PrP was detected in samples of the appendix of humans during the BSE pre-exposure era, suggesting that BSE was present even before BSE spread [11]. In addition, a 36-year-old British patient who was recently diagnosed with sporadic CJD was confirmed to have variant CJD by neuropathological examination after autopsy [12]. In Korea, the confirmed CJD rates by autopsy are considerably low at 1.6% among all CJD cases. Between 2016 and 2019, human prion diseases were mainly diagnosed by 14-3-3 protein detection in cerebrospinal fluid (CSF) and amplicon sequencing of the *PRNP* gene in blood-derived genomic DNA. However, since the sensitivity of 14-3-3 protein detection to diagnose variant CJD is under 50%, additional examinations are required to detect variant CJD in Korea [13].

In the present study, we investigated familial CJD-related germline mutations at codons 114, 178, 180, 183, 188, 196, 200, 203, 208, 210, 211, 232, and 238 of the *PRNP* gene in Korean patients after an appendectomy. In addition, we also investigated CJD-related genetic polymorphisms at codons 129 and 219 of the *PRNP* gene. Furthermore, we performed PrP^Sc^ detection using western blotting on the appendices of Korean patients.

## 2. Materials and Methods

### 2.1. Ethical Statements

All samples were provided with informed consent under institutional review board (IRB)-approved protocols. Brain samples from sporadic CJD patients and matched controls were provided by the University of Edinburgh. All procedures performed in the present study were approved by the IRB of Jeonbuk National University under approval numbers 2020-10-014 (brain) and 2019-03-010-001 (appendix), and they were conducted in accordance with the 1964 Helsinki Declaration and its later amendments. All of the samples and related data were anonymized prior to investigation.

### 2.2. Genomic DNA Extraction

Genomic DNA was isolated from 30 mg of each appendix sample using the BeadTM Genomic DNA Prep Kit (Biofact, Daejeon, Korea), following the manufacturer’s instructions. The quality of the genomic DNA was checked using a Nanodrop One spectrophotometer (Thermo Scientific, Waltham, MA, USA).

### 2.3. Genetic Analysis

To detect genetic polymorphisms and germline mutations in genomic DNA samples of Koreans, the *PRNP* gene was amplified through a polymerase chain reaction (PCR), using forward primer: 5′-CAACCGCTACCCACCTCAG-3′ and reverse primer: 5′-AGGACCATGCTCGATCCTCT-3′. The PCR mixture contained 1 µL (10–100 ng/µL) of genomic DNA, 10 pmol of forward and reverse primers, 2.5 µL of 10X *Taq* DNA polymerase buffer, 0.5 µL of a 0.2 µM dNTP mixture, 5 µL of 5X band helper, 0.25 µL *Taq* DNA polymerase, and sterile deionized water in a final volume of 25 µL. The PCR products were resolved by 1% agarose gels containing ethidium bromide. Target bands (564 bp) were purified using a Favor Prep GEL/PCR Purification Mini Kit (FAVORGEN, Pingtung City, Taiwan). The PCR products were directly sequenced with an ABI 3730 sequencer (ABI, Foster City, CA, USA), and the sequencing results were analyzed by Finch TV software (Geospiza, Inc., Seattle, WA, USA).

### 2.4. Protein Extraction

Tissue samples were homogenized in 10% volumes of RIPA lysis and extraction buffer (Thermo Fisher Scientific, Waltham, MA, USA) containing a complete protease inhibitor cocktail (Roche, Munich, Germany). The total protein concentration of the tissue homogenates was determined by BCA analysis (Thermo Fisher Scientific, Waltham, MA, USA).

### 2.5. Proteinase K Digestion

To detect PrP^Sc^ in the appendix samples, quantified tissue homogenates were digested by 50 µg/mL proteinase K for 1 h at 37 °C. The experimental conditions were selected by optimizing the protocol of PrP^Sc^ detection assay by loading gradient tissue-equivalent protein samples and adjusting the time/amount of protein K digestion. The proteinase K-treated samples were heated at 95 °C for 10 min in 5X sample buffer (Thermo Fisher Scientific, Waltham, MA, USA).

### 2.6. Western Blotting

The samples were loaded on a 12% sodium dodecyl sulfate (SDS) acrylamide gel. The proteins were separated at 100 V for 100 min and then transferred to nitrocellulose membranes (Amersham, Piscataway, NJ, USA) by an electrophoretic transfer system (Bio-Rad, Hercules, CA, USA) at 100 V for 100 min. The membranes were blocked using TBST (20 mM Tris-HCl, 150 mM NaCl, pH 7.6, 0.05% Tween-20) containing 5% skim milk for 2 h at room temperature. Subsequently, the membranes were incubated at 4 °C overnight with mouse monoclonal anti-PrP antibody (3F4, 1:200) (Enzo Biochem, Farmingdale, NY, USA). After washing in TBST several times, the membranes were incubated with mouse immunoglobulin G (IgG) (1:5000 in TBST) (Sigma–Aldrich, St. Louis, MA, USA) conjugated with horseradish peroxidase for 1 h at room temperature. After washing in TBST several times, the targeted protein was detected by chemiluminescence using ECL western blotting substrate (Thermo Fisher Scientific, Waltham, MA, USA).

## 3. Results

### 3.1. Subjects

A total of 86 appendix samples were collected from patients diagnosed with appendicitis by Presbyterian Medical Center in the Republic of Korea. Detailed information on the study population is described in Table 1. The sample size can also represent the total population of Korean with a 95% confidence level and a confidence interval of 6.34. There were 42 women and 44 men, and their mean age at the time of the appendectomy was 44.98 ± 24.07 years.

### 3.2. Investigation of Genetic Susceptibility Factors for CJD in Korean Patients with Appendectomy

We investigated genetic CJD-related germline mutations of the *PRNP* gene. The germline mutations of the human *PRNP* gene were not found in any of the patients with an appendectomy (Table 2). Besides these mutations, no *PRNP* mutations were found in patients with appendectomies.

We also investigated CJD-related genetic polymorphisms of the *PRNP* gene. Of the 86 patients with an appendectomy, 82 (95.35%) were homozygous for the M allele, 0 (0%) were homozygous for the V allele, and 4 (4.65%) were heterozygous at codon 129 of the *PRNP* gene, with an allele frequency of 0.9767:0.0233 (M:V). Among four patients with M129V heterozygous *PRNP* polymorphisms, two were male (Age, 18.5 ± 6.36) and two were female (Age, 62 ± 1.41). In addition, 76 (88.37%) were homozygous for the E allele, 0 (0%) were homozygous for the K allele, and 10 (11.63%) were heterozygous at codon 219 of the *PRNP* gene, with an allele frequency of 0.9419:0.0581 (E:K) (Table 2). Among 10 patients with E219K heterozygous *PRNP* polymorphisms, 8 were male (Age, 37.88 ± 27.37) and 2 were female (Age, 61.5 ± 3.54). Genotype and allele frequencies of the M129V and E219K were showed similar distributions to those of our previous studies obtained from a healthy Korean population (*p* > 0.05) [14,15].

### 3.3. CJD Inspection in Appendices of Koreans

**Figure 1:**
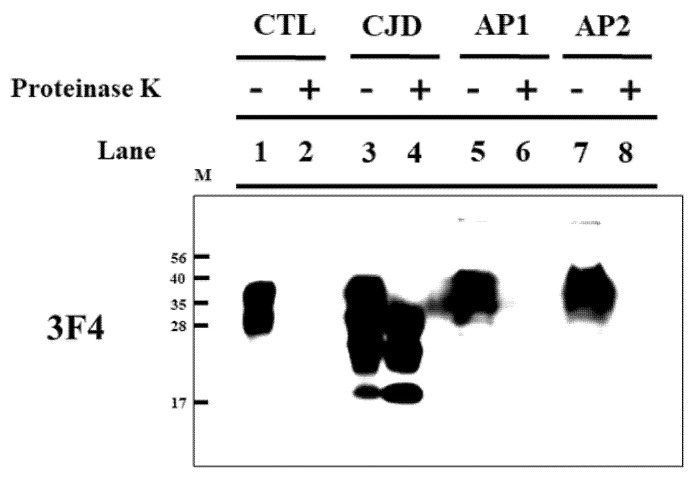
Western blotting detection of PrP^Sc^ in Korean appendices. Lane 1: Proteinase K-untreated frontal cortex from healthy controls. Lane 2: Proteinase K-treated whole brain from healthy controls. Lane 3: Proteinase K-untreated frontal cortex from a sporadic Creutzfeldt–Jakob disease (CJD) patient. Lane 4: Proteinase K-treated frontal cortex from a sporadic CJD patient. Lane 5: Proteinase K-untreated appendix from patient 1 with appendectomy (AP1). Lane 6: Proteinase K-treated appendix from patient 1 with appendectomy. Lane 7: Proteinase K-untreated appendix from patient 2 with appendectomy (AP2). Lane 8: Proteinase K-treated appendix from patient 2 with appendectomy. Bars on the left indicate the molecular size markers (in kilodaltons). CTL: a brain homogenate of healthy control as a negative control; CJD: a brain homogenate of sporadic CJD patient as a positive control; AP1: an appendix sample of patient 1; AP2: an appendix sample of patient 2; -: samples not treated with proteinase K; +: proteinase K-treated samples. 40 μg of protein was loaded in the lanes of healthy control and sporadic CJD patient. Over 100 μg of protein was loaded in each lane of Korean patients with appendectomy.

As positive and negative controls, the PrP^Sc^ band was investigated in brain homogenates of sporadic CJD patients and matched controls (Figure 1, Lanes 1–4). As expected, the PrP^Sc^ band in the brain homogenate of the matched control was not detected in the proteinase K-treated group (Figure 1, Lane 2), and the PrP^Sc^ band in the brain homogenate of sporadic CJD patients was detected in the proteinase K-treated group (Figure 1, Lane 4). The PrP^Sc^ band was investigated in the appendices of 86 patients (44 men and 42 women). Representative results are shown in Figure 1. The PrP^Sc^ band was not detected in any of the proteinase K-treated appendices (Lanes 6 and 8).

## 4. Discussion

Variant CJD is caused by oral ingestion of BSE-contaminated food, and prion agents are transported across the follicle-associated epithelium (FAE) through microfold cells in Peyer’s patch located in the small intestine. Subsequently, the prion agents are processed by follicular dendritic cells (FDCs) and then they start to accumulate in the spleen, lymph nodes, tonsils, and appendix [16,17]. In 95% of variant CJD patients, PrP^Sc^ is detected in the lymphoid tissues [18]. In the present study, we collected the appendix, one of the lymphoid tissues, from patients with appendicitis and then investigated PrP^Sc^ by western blotting. Notably, PrP^Sc^ was not detected in the appendix of any of the patients in this study (Figure 1).

Since variant CJD was first reported in the United Kingdom (UK) in 1996, it has been detected worldwide in more than 10 countries, including the UK, France, Ireland, USA, Canada, Italy, Saudi Arabia, Japan, Netherlands, Portugal, Spain, and Taiwan [19]. All variant CJD patients have a history of exposure to classical BSE [20]. The occurrence of classical BSE has been dramatically reduced due to the total ban on ruminant-derived meat and bone meal. However, a novel form of BSE—atypical BSE— still exists at a low-level, occurring in one in one million cattle worldwide. Atypical BSE is classified with H- and L-BSE by the molecular weight of unglycosylated PrP^Sc^ and it occurs in older cattle compared to classical BSE [21]. Atypical BSE has been reported in Asia, Europe, and the Americas, and it is thought to occur spontaneously [22,23,24,25,26,27]. In a previous study, H-type atypical BSE-infected cattle carrying the E211K germline mutation of the *PRNP* gene were reported in the USA in 2006, and this mutation is known to occur sporadically in H-type BSE [28,29]. Recently, high somatic mutation rates of E211K were detected in 3 Holstein cattle in Korea [30]. The E211K mutation may sporadically occur in H-type BSE and there is a high likelihood of atypical BSE anywhere in the world that cattle exist, with a common rate of one in one million cattle. Since more than four million cattle are raised in Korea (https://kostat.go.kr/ accessed on 5 May 2022), there is a possibility that a small number of atypical BSE will occur each year. In addition, the number and the rate of BSE inspections are relatively low in Korea; however, many Koreans consumed the byproducts of cattle, including intestine, bone, head, and blood. Thus, there is an increasing risk of a spread of variant CJD in Koreans.

In the UK, PrP^Sc^ was observed in two appendectomy samples (1970–1979) collected before the BSE outbreak [11]. In addition, PrP^Sc^ was detected in appendix sample cohorts collected in the periods 1941–1960 (733 per million) and 1961–1985 (412 per million) [31]. In Korea, variant CJD has not been reported thus far; however, there is a possibility of the existence of an asymptomatic variant CJD [32]. In Korea, 14-3-3 detection in CSF, which has a relatively low specificity and sensitivity for the diagnosis of variant CJD, has been used to diagnose CJD, and its confirmation rate through autopsy is very low. In previous studies, the detection of PrP^Sc^ in the brain was treated as a diagnostic method for confirmation of CJD, and the accumulation of PrP^Sc^ was observed in the brain of prion-infected mice prior to the onset of disease symptoms [33,34]. Although other diagnostic methods such as magnetic resonance imaging (MRI), electroencephalogram (EEG), and genetic testing of the *PRNP* gene have also been used for CJD diagnosis, the confirmation of PrP^Sc^ accumulation in the appendix is highly desirable to diagnose variant CJD in Korea since the PrP^Sc^ test on the appendix is a relatively comprehensive diagnostic method compared to brain autopsy.

To date, BSE has not been reported in Korea. However, despite atypical BSE sporadically occurring in several countries around the world, the number of BSE inspections is relatively low, and the byproducts of cattle were generally consumed in Korea. These reasons may possibly explain the presence of variant CJD in Koreans. Thus, we performed a preemptive inspection of variant CJD in Koreans, and we did not find PrP^Sc^ in all tested specimens. However, the assessment on PrP^Sc^ detection in Korean appendix samples was conducted in a relatively small number of samples. The absence of PrP^Sc^ in these samples seems reassuring, but it is uncertain because the sample size is very small. Thus, further evaluation in larger samples is needed in the future.

In addition, since the detection limit of PrP^Sc^ by western blotting is relatively low, PrP^Sc^ may not be detected. To resolve this limitation, further investigations using protein immunostaining, protein misfolding cyclic amplification (PMCA), real-time quaking-induced conversion (RT-QuIC), and bioassays in human PrP transgenic mice are needed in the future. In addition, brain tissue was used as positive and negative controls for PrP^Sc^ detection. However, the samples from 86 patients were not an accurate control because they were extracted from the appendix, and this is the limitation of the present study.

## 5. Conclusions

In conclusion, we investigated CJD-related genetic susceptibility factors, including polymorphisms and mutations, in 86 Korean patients undergoing appendectomy. In these Korean patients, no pathogenic mutations of the *PRNP* gene were found. In addition, among the 86 appendix samples, no samples showed protein K-resistant PrP. To the best of our knowledge, this was the first study of CJD-related genetic susceptibility factors with an evaluation of PrP^Sc^ in appendix samples obtained from Korean patients

## Figures and Tables

**Table 1:** Detailed information on the study population.

Characteristics		Samples
Number		86
Age (years)	Total	44.98 ± 24.07
	Male	39.95 ± 24.39
	Female	50.26 ± 22.85
Sex (*n*)	Male	44
	Female	42

**Table 2:** The genotype and the allele frequencies of polymorphisms and mutations of the prion protein gene (*PRNP*) in the study population.

	Genotype Frequency, *n* (%)			Allele Frequency, *n* (%)	
c.341G > T (G114V)	GG 86 (100)	GT 0 (0)	TT 0 (0)	G 172 (100)	T 0 (0)
c.385A > G (M129V)	AA 82 (95.35)	AG 4 (4.65)	GG 0 (0)	A 168 (97.67)	G 4 (2.33)
c.532G > A (D178N)	GG 86 (100)	GA 0 (0)	AA 0 (0)	G 172 (100)	A 0 (0)
c.538G > A (V180I)	GG 86 (100)	GA 0 (0)	AA 0 (0)	G 172 (100)	A 0 (0)
c.549A > G (T183A)	AA 86 (100)	AG 0 (0)	GG 0 (0)	A 172 (100)	G 0 (0)
c.563C > A (T188K)	CC 86 (100)	CA 0 (0)	AA 0 (0)	C 172 (100)	A 0 (0)
c.586G > A (E196K)	GG 86 (100)	GA 0 (0)	AA 0 (0)	G 172 (100)	A 0 (0)
c.598G > A (E200K)	GG 86 (100)	GA 0 (0)	AA 0 (0)	G 172 (100)	A 0 (0)
c.607G > A (V203I)	GG 86 (100)	GA 0 (0)	AA 0 (0)	G 172 (100)	A 0 (0)
c.623G > A (R208H)	GG 86 (100)	GA 0 (0)	AA 0 (0)	G 172 (100)	A 0 (0)
c.628G > A (V210I)	GG 86 (100)	GA 0 (0)	AA 0 (0)	G 172 (100)	A 0 (0)
c.631G > C (E211Q)	GG 86 (100)	GC 0 (0)	CC 0 (0)	G 172 (100)	C 0 (0)
c.655G > A (E219K)	GG 76 (88.37)	GA 10 (11.63)	AA 0 (0)	G 162 (94.19)	A 10 (5.81)
c.695T > G (M232R)	TT 86 (100)	TG 0 (0)	GG 0 (0)	T 172 (100)	G 0 (0)
c.712C > T (P238S)	CC 86 (100)	CT 0 (0)	TT 0 (0)	C 172 (100)	T 0 (0)

## Data Availability

All other data supporting the findings of this study are available from the corresponding author on reasonable request.
